# Supplementary material for: Temporal responses in sensorimotor cortex during hand movements
Source: PLoS One. 2026 May 7;21(5):e0347647. doi: 10.1371/journal.pone.0347647 (PMC13152139; doi:10.1371/journal.pone.0347647)
Supplement: S1 Text — (DOCX) [file pone.0347647.s001.docx]

**Text S1. Identification of frequency bands**

In this study, we aimed to empirically identify high- and low‑frequency bands that were consistently activated across participants. Frequency ranges were selected by visually inspecting the positive and negative clusters in the log‑transformed time-frequency plots for ‘thumb’ movements across participants P1–P8. Specifically, two time-frequency intervals were compared for significant differences using the ft_freqstatistics() function from the FieldTrip toolbox (in Matlab), which extracts positive and negative clusters that are consistent across channels (cluster alpha = 0.05; minimum number of contributing channels = 3). Permutation clustering was only computed for participants P4-P8 due to the absence of sufficiently long (3s) rest periods in the remaining participants. The time-frequency plots revealed two consistent clusters across channels and subjects: a negative cluster in the 5-30 Hz range and a positive cluster in the 60–100Hz range. Using permutation clustering, we confirmed that these clusters reflect significant differences between the movement and rest conditions across multiple channels for participants P4-P8. We therefore concluded that these clusters relate to movement initiation and execution, and we used the corresponding frequency ranges for subsequent analyses of neural activation patterns. Participant P10 was an individual with ALS and exhibited an atypical power spectrum compared with able-bodied participants, consistent with the observations reported by **Freudenburg et al. (2019)**. For this participant, the LFB was narrower than for P1-8 and the HFB 60-100Hz did not show significant activation. We therefore used the same LFB and HFB range employed in **Freudenburg et al. (2019)**.
