# Supplementary material for: Temporal responses in sensorimotor cortex during hand movements
Source: PLoS One. 2026 May 7;21(5):e0347647. doi: 10.1371/journal.pone.0347647 (PMC13152139; doi:10.1371/journal.pone.0347647)
Supplement: S1 Table — (DOCX) [file pone.0347647.s002.docx]

**Table S1. Channels included in the analysis.** Percentage of all included channels per participant with significant response to the task per frequency band and condition (thumb, index, little fingers, or finger movements).

|  | **THUMB** | | | | **INDEX** | | | | **LITTLE** | | | |
| --- | --- | --- | --- | --- | --- | --- | --- | --- | --- | --- | --- | --- |
|  | **alpha** | **beta** | **LFB** | **HFB** | **alpha** | **beta** | **LFB** | **HFB** | **alpha** | **beta** | **LFB** | **HFB** |
| **P01** | 100.0% | 100.0% | 100.0% | 91.7% | 100.0% | 100.0% | 100.0% | 100.0% | 100.0% | 100.0% | 100.0% | 95.8% |
| **P02** | 84.4% | 100.0% | 100.0% | 90.6% | 81.3% | 100.0% | 100.0% | 100.0% | 81.3% | 100.0% | 100.0% | 96.9% |
| **P03** | 94.8% | 100.0% | 100.0% | 72.4% | 100.0% | 100.0% | 100.0% | 75.9% | 100.0% | 100.0% | 100.0% | 87.9% |
| **P04** | 98.4% | 96.9% | 100.0% | 96.9% | 96.9% | 89.1% | 92.2% | 96.9% | 98.4% | 93.8% | 98.4% | 95.3% |
| **P05** | 60.3% | 57.1% | 66.7% | 96.8% | 65.1% | 68.3% | 74.6% | 95.2% | 50.8% | 47.6% | 55.6% | 96.8% |
| **P06** | 94.5% | 93.8% | 97.7% | 62.5% | 96.1% | 96.1% | 96.9% | 66.4% | 89.1% | 97.7% | 95.3% | 54.7% |
| **P07** | 99.2% | 100.0% | 100.0% | 82.5% | 100.0% | 100.0% | 100.0% | 80.0% | 98.3% | 100.0% | 100.0% | 82.5% |
| **P08** | 74.0% | 86.8% | 93.5% | 61.8% | 87.8% | 91.1% | 95.9% | 65.9% | 66.7% | 72.4% | 80.5% | 67.5% |
|  | **FINGERS** | | | |  |  |  |  |  |  |  |  |
| **P09** | 48.3% | 85.0% | 80.0% | 96.7% |  |  |  |  |  |  |  |  |
| **P10** | 12.5% | 0% | 0% | 62.5% |  |  |  |  |  |  |  |  |
