# Supplementary material for: Temporal responses in sensorimotor cortex during hand movements
Source: PLoS One. 2026 May 7;21(5):e0347647. doi: 10.1371/journal.pone.0347647 (PMC13152139; doi:10.1371/journal.pone.0347647)
Supplement: S2 Table — (DOCX) [file pone.0347647.s003.docx]

**Table S2. Timing difference between M1 and S1.** Difference between HFB mean onset time (in ms) for M1 (m_M1_) and S1 (m_S1_) channels is given per participant and finger. In addition, the proportion of M1 channels of all M1 and S1 channels in the quartile with the earliest onset points, per participant and finger.

|  | **THUMB** | | **INDEX** | | **LITTLE** | |
| --- | --- | --- | --- | --- | --- | --- |
|  | **m_M1_-m_S1_ (ms)** | **%M1/(S1+M1)** | **m_M1_-m_S1_ (ms)** | **%M1/(S1+M1)** | **m_M1_-m_S1_ (ms)** | **%M1/(S1+M1)** |
| **P01** | -0.050 | 67% | 0.060 | 75% | -0.112 | 100% |
| **P02** | -0.194 | 100% | -0.243 | 100% | -0.186 | 100% |
| **P03** | -0.198 | 100% | -0.050 | 33% | -0.173 | 67% |
| **P04** | -0.097 | 100% | -0.124 | 100% | -0.098 | 100% |
| **P05** | -0.105 | 100% | -0.117 | 100% | -0.149 | 100% |
| **P06** | -0.129 | 100% | -0.134 | 100% | -0.065 | 86% |
| **S07** | -0.248 | 100% | -0.168 | 100% | -0.071 | 100% |
| **P08** | -0.012 | 100% | 0.047 | 100% | 0.017 | 100% |
| **Mean** | **-129** | **96%** | **-91** | **89%** | **-105** | **94%** |
| **Std** | **80** | **12%** | **104** | **24%** | **66** | **12%** |
