# Supplementary material for: Temporal responses in sensorimotor cortex during hand movements
Source: PLoS One. 2026 May 7;21(5):e0347647. doi: 10.1371/journal.pone.0347647 (PMC13152139; doi:10.1371/journal.pone.0347647)
Supplement: S1 Fig — (DOCX) [file pone.0347647.s004.docx]

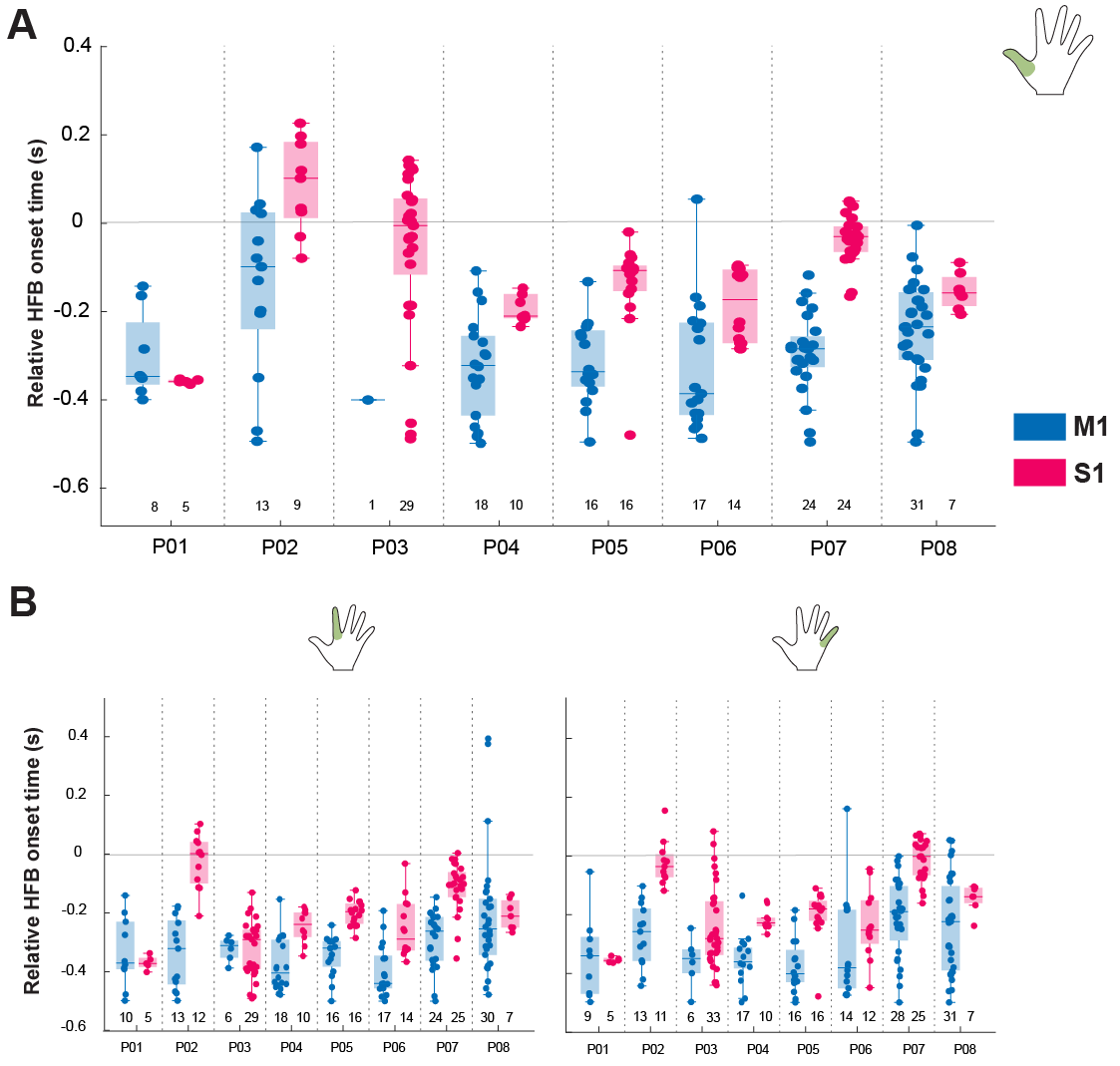


**Fig S1. Interpretation of neural onsets during execution of three fingers.** High-frequency band neural onset time (in seconds, s) for every M1 (blue points/boxplot) and S1 (red points/boxplot) channel, for the thumb (A), index (B, left panel) and little finger (B, right panel) and for all abled-bodied participants (P01-P08). The number of included channels per cortical region are indicated below each boxplot. Horizontal gray line (t = 0s) indicates the movement onset as detected with dataglove (MOM). Vertical dashed lines separate participants. Hand pictogram indicates which finger was moved. As expected, neural onsets in HFB occur in general before movement onset, both for M1 and S1 channels.
