# Supplementary material for: Temporal responses in sensorimotor cortex during hand movements
Source: PLoS One. 2026 May 7;21(5):e0347647. doi: 10.1371/journal.pone.0347647 (PMC13152139; doi:10.1371/journal.pone.0347647)
Supplement: S2 Fig — (DOCX) [file pone.0347647.s005.docx]

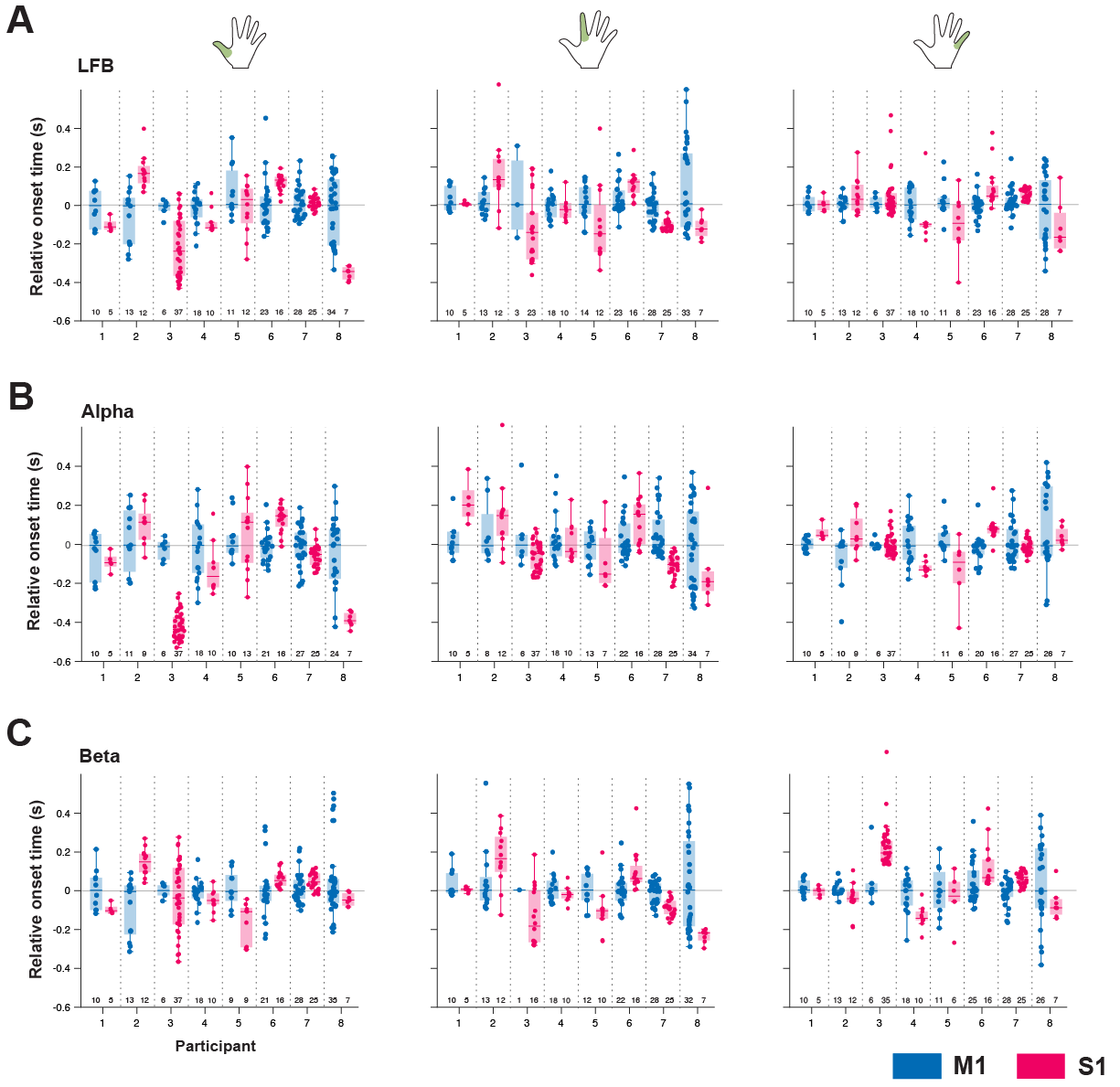


**Fig S2. Neural onsets during execution for low-frequency bands.** Neural onset time (in seconds, s) for every M1 (blue points/boxplot) and S1 (red points/boxplot) channel, for (A) the low-frequency band (LFB), (B) alpha band and (C) beta band for thumb (left panels), index (middle panels) and little (right panels) fingers, and for all abled-bodied participants (1-9 indicate P01-P09). To facilitate the visualization an effect line (gray solid line) was drawn between the M1 and S1 medians per participant. The number of included channels per cortical region are indicated below each boxplot. Horizontal gray line defines t = 0s (movement onset marker, MOM) and vertical dashed lines separate participants. Hand pictogram indicates which finger was moved.
