# Supplementary material for: Temporal responses in sensorimotor cortex during hand movements
Source: PLoS One. 2026 May 7;21(5):e0347647. doi: 10.1371/journal.pone.0347647 (PMC13152139; doi:10.1371/journal.pone.0347647)
Supplement: S2 Text — (DOCX) [file pone.0347647.s006.docx]

**Text S2. Movement onset detection based on muscle activity**

To better understand the onset times with respect to muscle activation and movement initiation, we compared the HFB neural onsets of P05 aligned with MOM with those aligned with EMGM **(Text S2 Figure)**. We recorded surface electromyography (EMG) measurements of the Abductor Policis Brevis in P05. EMG-onset markers (EMGM) were also manually extracted. For that line noise was removed from the EMG signal, then the signal was bandpass filtered between 30-300Hz and the root-mean-square (RMS) envelope with a 250ms window was computed. EMGM markers were identified visually by labeling the minimum point directly before the highest recorded peak of the EMG signal in the time interval between succeeding cues. We see that HFB pattern is preserved when using EMGM, with M1 channels activating prior to S1 channels. This result suggests a consistent offset between muscle activation and movement initiation and supports the use of MOM to interpret neural timing.


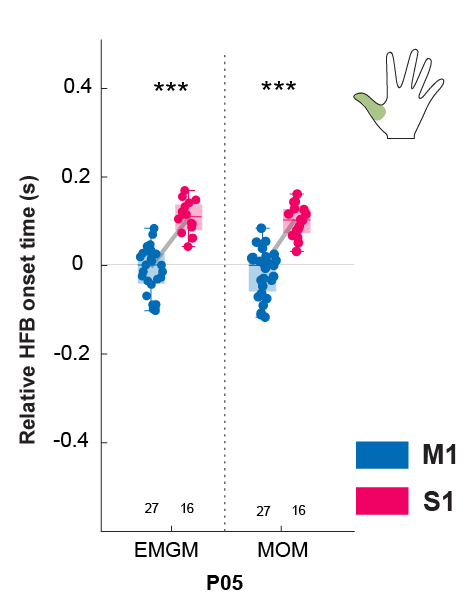


**Text S2 Figure. Trial alignment comparison between movement and EMG markers.** Relative HFB neural onsets (in seconds, s) in P06 using movement onset markers (MOM) detected via dataglove and EMG onset marker (EMGM), for the thumb finger. Vertical dashed line separates markers. M1 channels are indicated in blue, while S1 channels in red. The number of included channels per participant are indicated below each boxplot. Horizontal gray line (t = 0s) indicates the median onset time for M1 channels; all other onset times are displayed relative to this t=0s. Hand pictogram indicates which finger(s) the participant moved (solid line) or attempted (dashed line). Significant difference between M1 and S1 for EMGM and MO (post-hoc t-test) are indicated with *** p<0.001. P-values were Bonferroni corrected for the number of repetitions with more than 2 values per region (M1 and S1), that is N = 2.
