# Supplementary material for: Temporal responses in sensorimotor cortex during hand movements
Source: PLoS One. 2026 May 7;21(5):e0347647. doi: 10.1371/journal.pone.0347647 (PMC13152139; doi:10.1371/journal.pone.0347647)
Supplement: Attachment — Submitted filename: Response to reviewers.docx [file pone.0347647.s008.docx]

***Manuscript ID: PONE-D-25-59260***

We thank the editor and reviewers for their positive comments and suggestions. We address each comment separately below. Besides highlighting the changes in the manuscript, we have also copied the edited sections of the manuscript to this document to facilitate evaluation of the revision. The complete sections/sentences are in shown in *italic* and the edited/added text in ***bold-underlined***. We have also adjusted the manuscript style to PLOS ONE style.

This technically solid and important study investigates the temporal relationship between primary somatosensory cortex (S1) and primary motor cortex (M1) during both executed and attempted movements to reassess whether S1’s role extends beyond purely reafferent sensory processing. The authors analyze high-density ECoG recordings from eight participants with grids over the hand area of sensorimotor cortex (execution cohort), comparing the timing of responses in electrodes labeled M1 versus S1 across high-frequency band (HFB) and low-frequency band (LFB) activity, and then “verify” the timing pattern in two additional participants instructed to attempt movements with minimal sensory feedback. Their main finding is that HFB activity in M1 precedes S1 consistently across participants, even during attempted movement. LFB activity shows no consistent ordering. The authors interpret this as evidence that S1 is engaged around movement initiation, not merely a passive recipient of feedback.

Strengths
ECoG affords excellent temporal resolution and spatial coverage over peri-Rolandic cortex. The explicit contrast between executed and attempted movements targets the presence versus minimal presence of reafferent feedback. The band-specific analysis leverages the known link between HFB and local population spiking. If robustly quantified, a consistent M1-before-S1 lead in HFB, especially when overt feedback is minimized, does support an expanded view of S1 participation beyond classical sensory reception.

We thank the reviewer for the positive words and the thorough and constructive review. We have addressed each point separately below.

Weaknesses
However, several weaknesses limit the force of the conclusions as stated. First, the attempted-movement verification is based on only two participants, which might be underpowered for generalization. Second, concluding that S1 is “essential” for initiation overreaches the evidence: latency differences imply temporal engagement, not causal necessity (no perturbation, stimulation, or lesion-based tests are presented).

We agree with the reviewer that our sample of participants performing attempted movements is very limited. Accordingly, we have revised our statements and expanded the limitations section in the Discussion to explicitly address this constraint:

Discussion:

*“Last, in this study we examined the timing dynamics during the performance of actual movement in 8 participants. This inherently leads to the overlap of top-down motor commands and bottom-up sensory input and makes it impossible to draw causal conclusions of the origin of activation, especially pre-movement activations in S1.* ***We addressed this by comparing our results in two participants with minimal or no reafference (P09 and P10). Our preliminary findings show a similar pattern in HFB signals to that observed in able‑bodied participants. However, these observations need to be validated in a larger and more diverse cohort, including individuals with different neurological disorders. Indeed, several conditions, such as ALS, can lead to reduced grey‑matter thickness (Chen, 2013; Shen et al., 2016) and decreased HFB signals over M1 (Vansteensel et al., 2024), which may alter these dynamics****.*

***Looking ahead, emerging approaches combining ECoG with optogenetics offer a promising avenue for disentangling the respective contributions of M1 and S1. Such methods allow transient, region‑specific suppression or activation (Williams and Deisseroth, 2013; Yazdan-Shahmorad et al., 2018), enabling causal tests of how perturbing one area influences movement production and the neural responses of the other. Incorporating these techniques in future studies could provide a powerful way to isolate top‑down and bottom‑up components of movement‑related activity.”***

We also agree that our results do not demonstrate causality. We have therefore adjusted the Abstract to reflect that:

Abstract: *“These results call for an updated* ***view*** *of S1 beyond processing of sensory input, as* ***they suggest*** *S1* ***may*** *also* ***play a role*** *during movement initiation.”*

Recommendations for the authors

The movement-related HFB results in able-bodied participants are rigorous and convincingly show earlier onsets in M1 than S1. However, the central claim, an extended role of S1 during movement production beyond sensory input, rests primarily on the attempted-movement dataset, which includes only two participants and, for at least one of them (P10), sparse M1/S1 coverage with very few (2/1) responsive channels only for HFB activity and no channels for beta and LFB activity. Given this limited sample and coverage, the evidence for feedback-independent S1 engagement is suggestive but not robust. As it is written, the conclusion overreaches what the attempted-movement data can currently support. To strengthen the attempted-movement evidence, if possible, I recommend including data from at least one additional attempted-movement participant. If additional data are not feasible, I suggest tempering the claim to directional/indicative language rather than a strong verification of a feedback-independent S1 role.

We thank the reviewer for this suggestion. Unfortunately, we were not able to include additional data in the current manuscript. Accordingly, we have moderated our statements and added more cautious interpretation in the Discussion (see also previous point).

I suggest that the hypotheses paragraph can be improved by, for example, (i) defining what you mean by “neural activity features” and “timing” (e.g., onset vs peak, how derived from ECoG) and briefly mention how dataglove-detected movement onset is aligned; (ii) specifying the frequency ranges for HFB and LFB and why both are analyzed (e.g., HFB: local population firing; LFB(alpha, beta): preparatory and feedback-related dynamics?); (iii) avoiding the claim of “no feedback to S1” in attempted movement and instead stating how minimal reafference is verified; (iv) making clear you predictions and hypotheses.

We adjusted the last paragraph of the introduction to reflect the suggestions of the reviewer:

*“****To address the inconsistent findings on timing, we analyzed the onset latency of neural activity in M1 and S1 using ECoG, in relation to movement onset.*** *To separate somatosensory feedback from feedforward mechanisms to S1, we included two participants with no ability to move their hand who were implanted with electrodes on the affected hand region. Their movement attempt should activate the M1 but does result in* ***limited or no*** *feedback input to S1* ***due to amputation or paralysis****. We focus on the* *high-frequency band power* ***(60-100Hz)*** *given this is correlated with firing rates in neuronal populations as measured with ECoG (Ray et al., 2008; Miller, 2010) but include analyses of lower bands* ***(8-30Hz) due to their role in motor planning (Crone et al., 1998b). We expect electrodes over M1 to show earlier neural activation than those over S1, with both regions activating before movement onset****.”*

(Results Section 3.4: Executed and attempted movement): I suggest expanding this subsection, so the study’s central claim is quantitatively supported. For example, report subject-level statistics (e.g., latency differences) and electrode coverage and responsive-channel counts to contextualize P09 vs P10.

We now report subject-level statistic also in this section, as well as electrode coverage and responsive-electrode count, as follows:

*“Having established that HFB activity in M1 precedes that in S1 during executed finger movement, we* ***evaluated*** *whether this was also the case* ***for minimal or no reafference*** *during attempted movement (Figure 6). For two paralyzed participants P09 and P10 we aligned HFB neural responses using the GSM method.* ***P09 has 19 electrodes over M1 and 18 electrodes over S1, of which 19 and 15, respectively, showed a significant HFB response to the task. P1 had 3 electrodes covering M1 and 4 covering S1, of which only 2 channels over M1 and one over S1 showed a significant HFB response to the task.*** *Overall, the distribution of neural onset of participants P09 and P10 shows a similar pattern as in able-bodied participants, with an earlier HFB onset in M1* ***channels (P09: mean 0.13 ± 0.03s; P10: mean 0.22 ± 0.05)*** *compared to S1 channels* ***(P09: mean 0.19 ± 0.07s; P10: 0.23, only one channel)****. In both participants the first activating channel was located in M1, and for P09 78% of M1 channels fell in the earliest quartile.”*

(Discussion Section, para 1): After the sentence noting that Sun et al., 2015 compared “pairs of (sparsely placed) electrodes,” please add one explicit line that states your analysis unit and coverage and contrasts this with their pairwise, sparse sampling. This concrete clarification will make clear why methodological choices can flip apparent M1-S1 ordering.

We added this information to the discussion as follows:

“*These results are in direct contrast with those of Sun and colleagues who found earlier activation in S1 than M1 (Sun et al., 2015). However, this study compared the neural onset between* ***limited number*** *of sparsely placed electrodes* ***(spaced at least 1cm apart and often spreading beyond the hand region)****, which could lead to a biased interpretation of timing given that one can certainly find one electrode in S1 that activates before one electrode in M1.* ***In contrast, our analysis uses closely placed electrodes (3-4mm apart) within the hand area of M1 and S1, providing a much denser spatial coverage****.”*

The Discussion reads coherently and supports the main results. To make it even stronger before acceptance, I suggest light-touch refinements: avoid causal/“essential” phrasing; keep the attempted-movement inference explicitly preliminary, refresh a few citations with recent studies (last 5–7 years), and tighten band-specific messaging by presenting LFB results as inconclusive here while keeping HFB central. In the limitations paragraph, add one or two actionable next steps to underscore feasibility.

We have revised our discussion to address the comments from the reviewer. Regarding the references, we appreciate the importance to capture the latest reports on this topic. However, most of seminal work relevant to our study was published before 2020. To support our results in the discussion (section 4) we additionally added a study from 2020 that showed S1 single-unit spiking in a human with tetraplegia during imagined reach movement:

“*The directionality of information* ***agree with the findings from HFB signals of non-human primates (Umeda et al., 2019) and single unit spiking activity in humans (Jafari et al., 2020). Umeda and colleagues showed that although S1 becomes active before movement onset, increases in M1 activity consistently precede those in S1.*** *Their* ***results*** *support the idea that S1 activity during voluntary movement can be decoded from M1* ***prior*** *the arrival of proprioceptive and sensory inputs.* ***In addition, Jafari and colleagues demonstrated that S1 single units in a individual with tetraplegia encode imagined reaching movements and may contribute to the motor engagement even in the absence of sensation****. These results* ***stand in*** *direct contrast* ***to the findings*** *of Sun and colleagues, who reported earlier activation in S1 than* ***in*** *M1 (Sun et al., 2015).”*

Abstract (clarity and focus): Please streamline the abstract to center on the core, well-supported result: in HFB, M1 responses precede S1 across participants, with a preliminary observation that this pattern is also seen during attempted movement (n=2). I recommend omitting the LFB results from the abstract (or reducing to a brief “no consistent LFB effect”) since they are not shown in the main figures. Also, avoid causal language (e.g., “essential”). Briefly specify sample sizes (8 executed; 2 attempted) and what “timing” refers to (e.g., onset latency in HFB), and (minor) replace “prior or after” with “prior to or after.”

We thank the reviewer for the detailed suggestions. We adjusted the abstract as follows:

“*The role of primary somatosensory cortex (S1) in sensorimotor integration as limited to sensory processing has recently been challenged. In particular, it is unclear whether neural signals in S1 activate prior* ***to*** *or after the primary motor cortex (M1) in the presence and absence of* ***reafferent feedback****. Here we aim to gain a better understanding of the temporal dynamics between S1 and M1, and the underlying neural processes involved in movement execution. We compare the* ***onset latency*** *of S1 and M1 signals in eight able-bodied participants implanted with high-density electrocorticography (ECoG) grids over the hand region of the sensorimotor cortex for both low- and high-frequency band (LFB and HFB) signals.* ***Our results show a consistent activation of M1 electrodes before S1 electrodes across able-bodied participants for the HFB but no clear pattern for the LFB. Furthermore, we compared these results with two participants implanted with ECoG, who attempted movement with no or minimal reafference, showing similar effect in the HFB signals.*** *These results call for an updated* ***view*** *of S1 beyond processing of sensory input,* ***as they suggest S1 may also play a role during movement initiation****. Better understanding of the integration of M1 and S1 signals will be undoubtedly critical for the understanding of neuromuscular disorders and the development of neurotechnology*.”

I would consider moving the full Methods section to the end (keeping a brief Methods Summary in the main text) and restructuring as Introduction → Results → Discussion → Methods. This format improves readability and mirrors the structure used by many journals. This would make the narrative flow cleaner while keeping all technical information accessible.

We appreciate the reviewer’s suggestion and agree that moving the full Methods section to the end could improve readability. However, PLOS ONE guidelines require the Methods section to appear before the Results. Therefore, we have kept the original order.

Minor recommendations for the authors

(Section 1: Introduction): Consider rephrasing the following part to calibrate the strength of claims about S1 (e.g., implying it can drive movement independently of M1) and to resolve minor grammar issues: …with S1 being able to drive movements independently from M1 (Matyas et al., 2010), as well as being directly linked to motor learning (e.g., de Freitas Zanona et al., 2022) and movement planning (Gale et al., 2021) through cortical feedforward mechanisms that predict and integrates a efference copies of motor commands with incoming sensory signals (Arikan et al., 2021).

Thank for this suggestion. We have breakdown this large sentence in smaller sentence to improve readability:

*“In the last decades, however, a more multifaceted* ***view*** *of* ***S1’s role*** *in sensorimotor integration has emerged (Borich et al., 2015).* ***Evidence shows that*** *S1* ***is*** *able to drive movements independently from M1 (Matyas et al., 2010),* ***and is*** *directly linked to motor learning (e.g., de Freitas Zanona et al., 2022) and movement planning (Gale et al., 2021) through cortical feedforward mechanisms that predict and* ***integrate efference copies*** *of motor commands with incoming sensory signals (Arikan et al., 2021).”*

(Section 2.3: Task and behavioral response): Please briefly explain the rationale for the differing task parameters across participants (e.g., fingers tested, instructions, inter-trial timing) and indicate how these differences were accounted for in the analysis to ensure comparability.

We added a short rationale for use of different task parameters in section Task and behavioral response:

“***Please note that task paradigm and design for participants P1–P8 and P9–P10 were not identical, as the datasets were originally collected for other research purposes. Nevertheless, all participants were instructions to move their fingers.”***

And added this point to the limitation section in the discussion:

***“Another limitation is that task parameters differed across participants (e.g., fingers tested, specific instructions, and inter-trial timing). These differences were accounted for in the analysis to ensure comparability: all participants were instructed to move one or multiple fingers during active trials, which reliably activates the hand region of the sensorimotor cortex (e.g., Yousry et al., 1997; Branco et al., 2017; Schellekens et al., 2018). For alignment, we used movement onset for able-bodied participants and the GSM for participants P9–P10, ensuring all trials were referenced to a common point for comparison across channels and participants.”***

Revise main figure (Figure 4) for completeness. I suggest (i) including the attempted-movement participants (P09, P10) alongside P01–P08, (ii) reformat the plot with time on the x-axis and participants on the y-axis, and (iii) present three plots (A: thumb, B: index, C: little) with identical scales. This will make the M1→S1 timing pattern and its consistency across executed vs. attempted conditions immediately interpretable.

We appreciate the reviewer’s suggestions to improve readability of Figure 4. We have put the three fingers alongside using identical scales and flipped the x and y axis. Because data from attempted movement is very limited and only reflect preliminary results, we decided to keep these two figures separate and to tone down our conclusions.

(Section 4.1: Discussion): In the sentence “Here we show that HFB signals travel consistently from M1 to S1… whereas LFB… seems to be subject-specific,” please avoid causal wording, “travel” implies directed flow, and use timing language instead (e.g., “M1 HFB precedes S1”). In the following sentences on alpha/beta, state explicitly that the LFB results are inconclusive in your dataset, and maybe move the participant-specific band-definition suggestion to Limitations/Future work rather than presenting it as an explanation of the current results.

We have adjusted this section of the discussion accordingly:

***“****Here we show that HFB signals* ***in M1 precede those in S1****, both in the presence and absence of movement.* ***In contrast, LFB signals show no consistent tendency across subjects or fingers in able-bodied participants****.”*

(Section 4.4: Strengths & limitations): In the sentence “...two performed attempted movement and had therefore no sensory input,” please replace this with “minimal reafference.” If verification was performed, add a brief clause in the same paragraph indicating how (or whether) this was verified (e.g., EMG/kinematics, visual occlusion). As a general point, I recommend using “minimal reafference” rather than “no sensory input” throughout, as it better reflects the likely presence of unmeasured feedback.

We thank the reviewer from raising the issue of unmeasured feedback. While that is the case for P09, for whom we have no EMG verification of residual arm movement, for P10 we have medical confirmation of no arm/hand EMG activity produced during hand movement which was in line with the late-stage of the disease progression and the very low ALS-frs score for the participant at the time of implant (score of 2).

As suggested, we substituted where applicable, the concept of ‘sensory input’ by (minimal or no) reafference and added to the methods a sentence justifying lack of muscle activity in P10:

Section Participants: ***“P10 had a ALS functional rating scale score of 2 with, no visible reafference as measured with electromyography on the hand and arm.”***

This manuscript presents an important and ambitious investigation of the temporal dynamics between the primary motor cortex (M1) and primary somatosensory cortex (S1) using high-density ECoG recordings during both executed and attempted finger movements. The study leverages a rare and valuable dataset, including two participants without sensory input, and addresses a fundamental question in sensorimotor neuroscience with clear relevance for understanding motor control, neuromuscular disorders, and neurotechnology development. The results particularly the consistent finding that high-frequency band (HFB) activity in M1 precedes that in S1 are intriguing and potentially impactful. However, several essential aspects of the Methods require clearer description and stronger justification before the manuscript can be considered robust and reproducible. The major comments are outlined below.

We thank the reviewer for their suggestions. We address each point separately below.

1. Although the electrode localization procedure has been described in previous publications, the current manuscript should still provide a brief explanation of the algorithm and specify which software tools or custom code were used. Even if the method follows prior work, readers need enough methodological detail to understand and reproduce the electrode coregistration workflow. A short description of the steps and software would greatly improve clarity.

We added a short description of the method in section Electrode localization and labelling:

“*Electrode localization for* ***P01-P08 and P10*** *(****Figure 1****) was performed by co-registering high resolution post-implantation* ***3D-****Computerized Tomography (CT) scans (Philips Tomoscan SR7000, Best, The Netherlands;* ***voxel size 0.49x0.49xT mm, with T ranging from 0.45 to 1mm****) with preoperative T1-weighed anatomical scans on a 3T Magnetic Resonance system (Philips 3T Achieva, Best, The Netherlands;* ***isotropic voxels size of 1mm****).* ***Followed by the detection of electrodes on the CT, computing each their center of mass, and then projecting them onto the individual freesurfer cortical surface while correcting for brain shift using local grid geometry (Branco et al., 2017b; Hermes et al., 2010).***”.

2. It would greatly improve clarity if the authors could include a methodological flowchart summarizing the full analysis pipeline. A visual overview of the key steps—such as electrode localization, channel selection, trial alignment, feature extraction, and onset detection—would help readers better understand the entire procedure and how each component fits together.

We thank the reviewer for this suggestion. We provide an overview of the methods in the new Figure 2 panel A.

3. It is not clearly described how the MRI beta maps were obtained. The authors should indicate what task paradigm was used, which software package processed the fMRI data, and which MRI sequences were acquired. Important acquisition parameters for both T1-weighted structural imaging and fMRI should be included. In addition, clarification is needed regarding how and when the fMRI data were collected prior to ECoG implantation.

In the present study we did not include any fMRI data. Instead, we used a previous published study from our lab to estimate the expected functional hand region in our participants. For that we used a toolbox called Cgrid to map all participants electrode coordinates to a cartesian common space, which was also used in previous fMRI study. Details about the fMRI data acquisition and preprocessing can be found in Bruurmijn et al 2020 (Figure 3).

We adjusted our methodology in section Electrode localization and labelling to clarify this point:

“*For participants P01-P08 and P10, we identified electrodes that were strictly over the hand-knob region by constraining the coordinates of the electrodes that fall within a region expected to have a response to hand movement.* ***The coordinate constraint was determined by mapping the subject electrode coordinates to a common space using a Cgrid method (***[***https://github.com/mathijsraemaekers/Cgrid-toolbox***](https://github.com/mathijsraemaekers/Cgrid-toolbox)***)*** *that converts individual surface sensorimotor spaces to a 2D standard cartesian grid* ***of 40x80 tiles*** ***(Bruurmijn et al., 2020; Raemaekers et al., 2020)****.* ***Then we identified a minimum and maximum y-coordinated (***$\boldsymbol{41\leq y\leq65}$***) based on the functional Magnetic Resonance Imaging beta-maps of 20 healthy participants performing a hand localizer task reported in (Bruurmijn et al., 2020)****.”*

4. The manuscript does not provide sufficient information about the CT acquisition parameters. Key CT details such as slice thickness, voxel dimensions, scanner settings, and reconstruction methods should be reported, as these directly affect the accuracy of electrode localization.

In this study, CT (and MRI) parameters were determined by clinical need. In sum, it consisted of a 3D-CT scan with anisotropic voxel size of 0.49x0.49xT, where T ranged from 0.45 to 1 mm. This difference varied based on availability of one or multiple CT scanners at the UMC Utrecht. We added the voxel details to the manuscript. More details about the CT can be found in Branco et al. 2017b:

*“Electrode localization for P01-P08 and P10 (Figure 1) was performed by co-registering high resolution post-implantation* ***3D-****Computerized Tomography (CT) scans (Philips Tomoscan SR7000, Best, The Netherlands;* ***voxel size 0.49x0.49xT mm, with T ranging from 0.45 to 1mm****) with preoperative T1-weighed anatomical scans on a 3T Magnetic Resonance system (Philips 3T Achieva, Best, The Netherlands;* ***isotropic voxels size of 1mm****).”*

5. The rationale for defining the high-frequency band (HFB) as 60–100 Hz is not clearly justified. It would be important to state whether other commonly used high-gamma ranges (e.g., 60–150 Hz or 60–200 Hz) were tested. Furthermore, the manuscript should explain why HFB is defined as 60–100 Hz for P09 but 30–100 Hz for P10. Because the conclusions rely heavily on HFB timing, a clearer justification or data-driven selection of frequency bands is necessary.

We thank the reviewer for raising this point. We were interested in empirically finding HF and LF bands that were consistently activated across participants. In our study the frequency ranges were selected by visual inspection of the positive and negative clusters in the log-transformed time-frequency plots for the thumb movement across P1-P8. Two time-frequency intervals were compared for significant difference using the ft_freqstatistics() function in Fieldtrip toolbox, which extracts positive and negative clusters that are consistent across channels (cluster alpha=0.05 and minimum number of consistent channels=3). Permutation clustering was only computed for sub-4-8, due to lack of 3s rest periods in the remaining participants. Time-frequency plotting shows two different clusters, one negative in the frequency range 5 to 30 Hz (blue) and a positive cluster (red) in the frequency band 60 to 100Hz (see figure below). This pattern is consistent across channels and subjects. Using permutation clustering, we were able to show that these clusters reflect a significant difference between the movement and the rest condition in multiple channels for P4-8. Therefore, we concluded that they are related to movement initiation and execution and used the corresponding frequency ranges for further analysis of neural activation patterns.

Regarding P10, this subject was an ALS subject which showed atypical power spectrum compare with able-bodied participants (as described in Freudenburg et al 2019). For that subject the band 60-100 Hz did not show significant activation, and therefore we chose to use the same band as in the study of Freudenburg 2019 (30-100 Hz), which capitalizes on the broadband increase in power.

We added the motivation behind the selection of HFB and LFB to Supplementary Materials and refer to it in section 2.6.

Figure: Example of time-frequency-clustering for P4 for all channels separately (upper figure left) and the corresponding permutation clustering plot (upper figure right). The third plot below shows the same clusters as in the time-frequency-plot across but in the mean of all channels.

6. The procedure for performing the one-sided paired t-tests on electrodes showing significant task responses is not sufficiently explained. It is unclear whether the t-tests were conducted per channel, per participant, or across channels. The manuscript should specify the p-value threshold used for significance, and if multiple t-tests were performed, it is important to clarify whether any correction for multiple comparisons (e.g., Bonferroni correction or FDR) was applied. Without this information, it is difficult to assess the statistical rigor of the significance testing.

We have adjusted the text in section Electrodes with significant response to the task to add details about the statistical testing:

*“****For each task and participant (P1-8), significant channels*** *were identified by means of a one-sided paired t-test* ***(p<0.05, no multiple comparison correction)*** *between the mean pre-marker period (-1 to -0.5s) and a post-marker interval (0 to 0.5s) per trial.”*
